# Combination genetic signature stratifies lower-grade gliomas better than histological grade

**DOI:** 10.18632/oncotarget.4928

**Published:** 2015-08-11

**Authors:** Aden Ka-Yin Chan, Yu Yao, Zhenyu Zhang, Zhifeng Shi, Liang Chen, Nellie Yuk-Fei Chung, Joseph Shu-Ming Liu, Kay Ka-Wai Li, Danny Tat-Ming Chan, Wai Sang Poon, Ying Wang, Liangfu Zhou, Ho-Keung Ng

**Affiliations:** ^1^ Department of Anatomical and Cellular Pathology, Chinese University of Hong Kong, Shatin, Hong Kong; ^2^ Shenzhen Research Institute, The Chinese University of Hong Kong, Shenzhen, China; ^3^ Neurosurgery Division, Department of Surgery, Chinese University of Hong Kong, Shatin, Hong Kong; ^4^ Department of Neurosurgery, Huashan Hospital, Fudan University, Shanghai, China; ^5^ Department of Neuropathology, Huashan Hospital, Fudan University, Shanghai, China

**Keywords:** Pathology Section, glioma, IDH1/2, 1p/19q, TERT, EGFR

## Abstract

We studied if combination genetic signature potentially stratifies lower-grade gliomas better than histology by investigating 214 lower-grade gliomas for *IDH1/2* and *TERT*p mutations, 1p/19q codeletion and *EGFR* amplification as to their impact on prognostication. Prognostic association of grading was independent of other prognostic variables including age, histological type, *IDH1/2*, 1p/19q and *TERT*p status. No single marker, including *IDH1/2*, superseded grading in prognostication, indicating grading was still a very important tool. Prognosis was most favorable in 31.7% of patients with *IDH1/2* mutation and either 1p/19q codeletion or *TERT*p mutation (IDHmut-OT), intermediate in 45.8% of patients with *IDH1/2* mutation only (IDHmut) and 16.9% of patients without any of the alterations (IDHwt), and poorest in 5.6% of patients with wild-type *IDH1/2* and either *TERT*p mutation or *EGFR* amplification (IDHwt-ET). Our results suggested not all *IDH1/*2 wild-type lower-grade gliomas are aggressive and additional biomarkers are required to identify glioblastoma-equivalent tumors. Multivariate analysis revealed independent prognostic values of grading and genetic signature. Grade II IDHwt-ET gliomas exhibited shorter survival than *IDH1/2* mutated grade III gliomas, suggesting combination genetic signature potentially superseded grading in prognostication. In summary, biomarker-based stratification is useful in the diagnosis and prognostication of lower-grade gliomas, and should be used together with grading.

## INTRODUCTION

Lower-grade glioma is a diversified group of infiltrative brain tumors comprising WHO grades II and III astrocytomas, oligodendrogliomas and mixed oligoastrocytomas. The tumors exhibit wide range of biological and clinical heterogeneity and remain as one of the major challenges in clinical practice in neuro-oncology. One of which is the interobserver variability arising from the morphology-based classification of diffuse gliomas which leads to suboptimal diagnostic reproducibility affecting both histological typing and grading [1–4]. Accurate differentiation of histological grade is crucial in clinical management of lower-grade gliomas not only for prognostic estimation but treatment guidance [5–9]. In most places, lower-grade gliomas are treated by maximal resection and high grade tumors need adjuvant therapy in most regimens [5, 9]. Such differentiation is of particular importance in surgical biopsy and yet there is major interobserver discrepancy [4]. Therefore, more objective and reproducible markers which can supersede histological grade are needed for clinical stratification of lower-grade gliomas. In order to improve the diagnostic and prognostic accuracy and facilitate therapeutic formulation, molecular markers will be supplemented into the coming WHO classification of lower-grade glioma so as to provide a more objective and precise tumor classification system [10]. *Isocitrate dehydrogenase 1* (*IDH1*) mutation and chromosome 1p/19q codeletion represent the most important molecular markers with clinical implications in lower-grade gliomas. Both of the markers are strong favorable prognostic factors in lower-grade gliomas as shown in the literature [11–18], and their predictive values to PCV (procarbazine, CCNU and vincristine) chemotherapy in oligodendroglial tumors have also been recently revealed in clinical trials [11, 12, 17]. Promoter mutation of *telomerase reverse transcriptase* (*TERT*p) is another emerging molecular marker which can stratify lower-grade gliomas and glioblastomas into prognostic subgroups, particularly in combination with *IDH1* mutation [19–24]. The activating mutation is associated with 1p/19q codeletion and oligodendroglial histology in the presence of *IDH1* mutation and identifies a subset of aggressive astrocytic tumors lacking *IDH1* mutation, highlighting its potential diagnostic and prognostic impact in lower-grade gliomas [19, 20, 23, 25, 26]. Incorporation of molecular markers into tumor classification not only provides additional prognostic information but potential influence on the histological grade of tumor diagnosis. For example, *IDH1* mutation has been reported to exert a greater prognostic relevance in glioblastoma and anaplastic astrocytoma than histological grade, indicating that molecular markers might potentially supersede histological grade in tumor classification [27]. The influence of molecular markers in the prognostic value of histological grade in lower-grade gliomas, however, remains under-investigated. In this study, we aim to examine a panel of molecular markers including *IDH1/2* mutation, 1p/19q codeletion, *TERT*p mutation and *EGFR* amplification as to their impact on the prognostic value of histological grade in a cohort of lower-grade gliomas. Our study reveals that combination genetic signature potentially supersedes histological grade in prognostic classification of lower-grade gliomas and has important clinical implication in refining the diagnostic and prognostic accuracy of grades II and III gliomas.

## RESULTS

### Cohort characteristics

**Figure 1:**
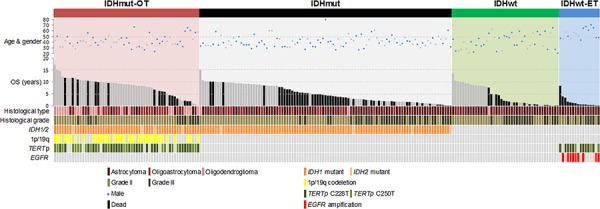
Clinical and molecular data of 214 lower-grade gliomas in the study 1p/19q codeletion occurred exclusively in *IDH1/*2 mutated gliomas (*p* < 0.0001, Chi square test). *EGFR* amplification was mutually exclusive with *IDH1/2* mutation (*p* < 0.0001, Fisher's exact test) and associated with *TERT*p mutation within the *IDH1/2* wild-type gliomas (*p* = 0.002, Fisher's exact test). Tumors were assigned into genetic subgroups based on combinations of status of *IDH1/2* mutation, 1p/19q codeletion, *TERT*p mutation and *EGFR* amplification. IDHmut-OT gliomas were defined as tumors harboring *IDH1/2* mutation and either 1p/19q codeletion or *TERT*p mutation. IDHmut gliomas were defined as tumors harboring *IDH1/2* mutation only and without 1p/19q codeletion nor *TERT*p mutation. IDHwt gliomas were defined as tumors harboring none of the molecular markers. IDHwt-ET gliomas were defined as *IDH1/2* wild-type tumors with either *TERT*p mutation or *EGFR* amplification. The cases were sorted by overall survival within each genetic signature subgroup. OS, overall survival.

Table 1 and Fig. 1 summarize the clinical and molecular data of the cohort. Seventy-eight cases from Prince of Wales Hospital (Hong Kong) and 136 cases from Huashan Hospital (Shanghai) formed the basis of the cohort. The cohort comprised 142 grade II diffuse gliomas which included 86 diffuse astrocytomas, 18 oligodendrogliomas and 38 oligoastrocytomas, as well as 72 grade III anaplastic gliomas which included 63 anaplastic astrocytomas, 6 anaplastic oligodendrogliomas and 3 anaplastic oligoastrocytomas. Male to female ratio of the cohort was 1:0.69. The mean and median ages were 41.9 and 41 years (range 20 to 79), respectively. Operation data was available in 199 of 214 (93%) patients and adjuvant treatment data was available in 188 of 214 (87.9%) patients. Total resection was performed in 83 of 137 (60.6%) grade II tumors and 29 of 62 (46.8%) grade III tumors (*p* = 0.07). Radiotherapy was given in 95 of 128 (74.2%) grade II tumors and 47 of 60 (78.3%) grade III tumors (*p* = 0.503). Chemotherapy was given in 83 of 128 (64.8%) grade II tumors and 34 of 60 (56.7%) grade III tumors (*p* = 0.281). Survival data was available in 214 patients. The median follow-up and median overall survival were 9.2 years and 8.4 years, respectively.

**Table 1:** Summary of clinical and molecular data of lower grade gliomas in the study

	Grade II *n* = 142	Grade III *n* = 72	All tumors *n* = 214
Gender (Male /Female)	79/63	48/24	127/87
Age (Mean / median / range)	40.4 / 39 (21–79)	44.9 / 44.5 (20–70)	41.9 / 41 (20–79)
Histological type			
Astrocytomas	86 (60.6%)	63 (87.5%)	149 (69.6%)
Oligodendrogliomas	18 (12.7%)	6 (8.3%)	24 (11.2%)
Oligoastrocytomas	38 (26.8%)	3 (4.2%)	41 (19.2%)
Operation			
Total resection	83 (58.5%)	29 (40.3%)	112 (52.3%)
Non-total resection	54 (38%)	33 (45.8%)	87 (40.7%)
Not available	5 (3.5%)	10 (13.9%)	15 (7%)
Adjuvant therapy			
Radiotherapy only	22 (15.5%)	16 (22.2%)	38 (17.8%)
Chemotherapy only	10 (7%)	3 (4.2%)	13 (6.1%)
Both radiotherapy and chemotherapy	73 (51.4%)	31 (43.1%)	104 (48.6%)
No	23 (16.2%)	10 (13.9%)	33 (15.4%)
Not available	14 (9.9%)	12 (16.7%)	26 (12.1%)
*IDH1/2*			
Mutant	110 (77.5%)	46 (63.9%)	156 (72.9%)
Wild-type	32 (22.5%)	26 (36.1%)	58 (27.1%)
1p/19q			
Codeleted	35 (24.6%)	3 (4.2%)	38 (17.8%)
Non-codeleted	107 (75.4%)	69 (95.8%)	176 (82.2%)
*TERT*p			
Mutant	41 (28.9%)	20 (27.8%)	61 (28.5%)
Wild-type	101 (71.1%)	52 (72.2%)	153 (71.5%)
*EGFR*			
Amplified	6 (4.2%)	3 (4.2%)	9 (4.2%)
Non-amplified	136 (95.8%)	69 (95.8%)	205 (95.8%)
Genetic signature			
IDHmut-OT	45 (31.7%)	12 (16.7%)	57 (26.6%)
IDHmut	65 (45.8%)	34 (47.2%)	99 (46.3%)
IDHwt	24 (16.9%)	18 (25%)	42 (19.6%)
IDHwt-ET	8 (5.6%)	8 (11.1%)	16 (7.5%)

*n*, number of case; IDHmut-OT, *IDH1/2* mutated and either 1p/19q codeleted or *TERT*p mutated; IDHmut, *IDH1/2* mutated only; IDHwt, *IDH1/2* wild-type, *TERT*p wild-type and *EGFR* non-amplified; IDHwt-ET, *IDH1/2* wild-type and either *TERT*p mutated or *EGFR* amplified.

### *IDH1/2* mutation

*IDH1/2* mutations were found in 156 of 214 (72.9%) of tumors examined, including 59 of 86 (68.6%) diffuse astrocytomas, 17 of 18 (94.4%) oligodendrogliomas, 34 of 38 (89.5%) oligoastrocytomas, 37 of 63 (58.7%) anaplastic astrocytomas, 6 of 6 (100%) anaplastic oligodendrogliomas and 3 of 3 (100%) anaplastic oligoastrocytomas. Regarding histological grade, *IDH1/2* mutations were detected in 110 of 142 (77.5%) grade II tumors and 46 of 72 (63.9%) grade III tumors. Among the 156 *IDH1/2* mutated tumors, *IDH1* and *IDH2* mutations were mutually exclusive and were observed in 148 (94.9%) and 8 (5.1%) cases, respectively. 147 of 148 (99.3%) *IDH1* mutations were IDH1-R132H and 6 of 8 (75%) *IDH2* mutations were IDH2-R172K. Other mutation subtype observed included one case of IDH1-R132C, one case of IDH2-R172M and one case of IDH2-R172G.

### Chromosome 1p/19q codeletion

Chromosome 1p/19q codeletion was detectable in 38 of 214 (17.8%) of tumors examined, including 6 of 86 (7%) diffuse astrocytomas, 11 of 18 (61.1%) oligodendrogliomas, 18 of 38 (47.4%) oligoastrocytomas and 3 of 6 (50%) anaplastic oligodendrogliomas. Twenty-five percent of grade II tumors and 4.2% of grade III tumors harbored 1p/19q codeletion. 1p/19q codeletion was found exclusively in *IDH1/2* mutated tumors (*p* < 0.0001). According to histological tumor type, 1p/19q codeletion was observed in 14 of 24 (58.3%) oligodendrogliomas, 18 of 41 (43.9%) oligoastrocytomas and 6 of 149 (4%) astrocytomas (*p* <0.00001) (Fig. 1).

### *TERT* promoter mutation

*TERT*p mutation was identified in 61 of 214 (28.5%) tumors examined, including 41 of 142 (28.9%) grade II gliomas and 20 of 72 (27.8%) grade III gliomas. Among grade II tumors, 13 of 86 (15.1%) diffuse astrocytomas, 13 of 18 (72.2%) oligodendrogliomas and 15 of 38 (39.5%) oligoastrocytomas harbored *TERT*p mutation. Among grade III tumors, mutation was found in 12 of 63 (19%) anaplastic astrocytomas, 6 of 6 (100%) anaplastic oligodendrogliomas and 2 of 3 (66.7%) anaplastic oligoastrocytomas, respectively. Thirty-eight (62.3%) mutated tumors showed C228T mutation and 23 (37.7%) mutated tumors showed C250T mutation, of which the two mutation subtypes were mutually exclusive.

### *EGFR* amplification

In all lower-grade gliomas examined for *EGFR* FISH analysis, *EGFR* amplification was found in 6 of 86 (7%) diffuse astrocytomas and 3 of 63 (4.8%) anaplastic astrocytomas, giving an overall amplification frequency of 4.2% (9/214). *EGFR* amplification was exclusively found in tumors with wild-type *IDH1/2* (*p* < 0.0001) and showed co-occurring association with *TERT*p mutation in *IDH1/2* wild-type tumor subset (*p* = 0.002).

### Survival analysis (Individual variable)

**Figure 2:**
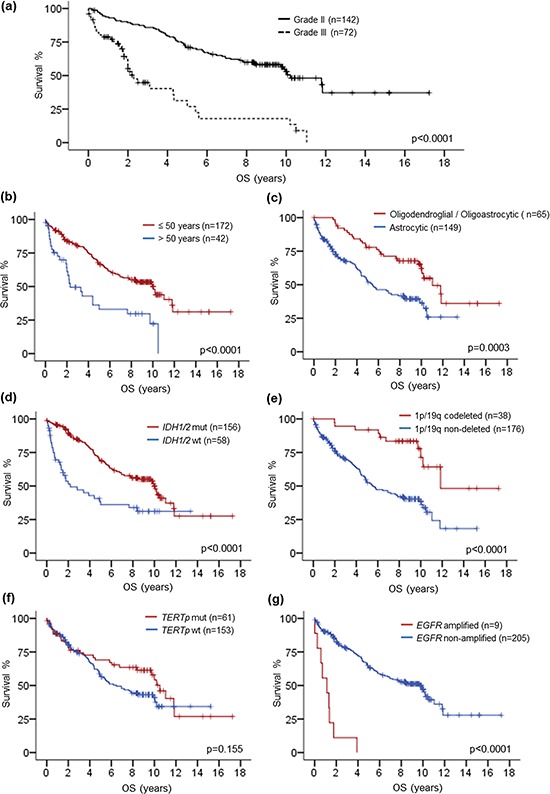
Kaplan-Meier survival analysis by histological grade, age, histological type, IDH1/2 mutation, 1p/19q codeletion, TERTp mutation and EGFR amplification Longer overall survival was associated with grade II gliomas **a.** patients at 50 years or younger **b.** oligodendroglial or oligoastrocytic tumors **c.** *IDH1/2* mutated gliomas **d.** 1p/19q codeleted gliomas **e.** and *EGFR* non-amplified gliomas **g.** *TERT*p mutated gliomas trended to longer overall survival **f.** OS, overall survival; mut, mutant; wt, wild-type.

We evaluated the survival data according to histological grade, patient age, histological type and various molecular markers by univariate analysis (Table 2, Fig. 2a to 2g). Patients with grade II gliomas had longer overall survival than grade III gliomas (*p* < 0.0001) (Fig. 2a). The median overall survival was 10.2 years for grade II tumors and 2.3 years for grade III tumors. Patients of 50 years or younger had longer overall survival than those above 50 years (*p* < 0.0001) (Fig. 2b). Stratifying the cohort according to histological type, tumors with oligodendroglial or oligoastrocytic histology showed longer overall survival than tumors with astrocytic histology (*p* = 0.0003) (Fig. 2c). Regarding molecular markers, patients with *IDH1/2* mutation (*p* < 0.0001) and 1p/19q codeletion (*p* < 0.0001) had more favorable overall survival than those without the markers (Fig. 1d and 1e). Patients with *TERT*p mutated tumors showed trend of longer overall survival than those with *TERT*p wild-type tumors (Fig. 2f). Tumors with *EGFR* amplification exhibited very short overall survival comparing to tumors without the gene amplification (*p* < 0.0001) (Fig. 2g).

**Table 2:** Univariate analysis of clinical and molecular variables

	*n*	HR	[95% CI]	Median OS (years)	*p*
Histological grade					
Grade II	142	1		10.2	<0.0001
Grade III	72	4.045	[2.629–6.223]	2.3	
Age					
≤50 years	172	1		10	<0.0001
>50 years	42	2.43	[1.549–3.812]	2.3	
Histological type					
Astrocytic	149	2.266	[1.445–3.555]	5.5	0.0003
Oligodendroglial / Oligoastrocytic	65	1		11	
*IDH1/2*					
Mutant	156	0.431	[0.286–0.649]	10.1	<0.0001
Wild-type	58	1		2.2	
1p/19q codeletion					
Codeleted	38	0.274	[0.142–0.529]	11.8	<0.0001
Non-codeleted	176	1		5.6	
*TERT*p					
Mutant	61	0.73	[0.472–1.128]	10.5	0.155
Wild-type	153	1		6.5	
*EGFR*					
Amplified	9	9.082	[4.373–18.863]	1.1	<0.0001
Non-amplified	205	1		9.9	
Genetic signature					
IDHmut-OT	57	1		11.8	<0.0001
IDHmut	99	2.863	[1.643–4.989]	5.9	
IDHwt	42	2.883	[1.498–5.547]	5	
IDHwt-ET	16	17.65	[8.603–36.208]	0.6	

*n*, number of case; HR, hazard ratio; 95% CI, 95% confidence interval; OS, overall survival; IDHmut-OT, *IDH1/2* mutated and either 1p/19q codeleted or *TERT*p mutated; IDHmut, *IDH1/2* mutated only; IDHwt, *IDH1/2* wild-type, *TERT*p wild-type and *EGFR* non-amplified; IDHwt-ET, *IDH1/2* wild-type and either *TERT*p mutated or *EGFR* amplified.

**Figure 3:**
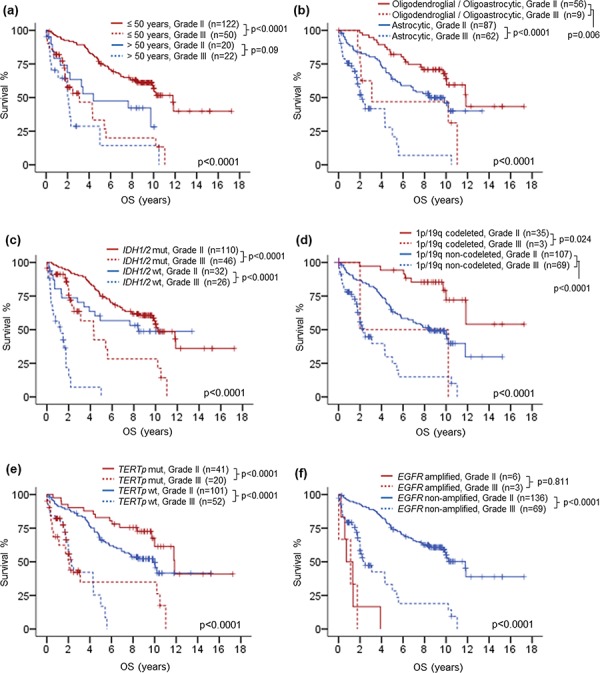
Kaplan-Meier survival analysis by co-evaluating histological grade with clinical and molecular variables Histological grade showed prognostic relevance in patients of 50 years or younger and trend in patients above 50 years **a.** Prognostic relevance of histological grade was demonstrated in both astrocytic gliomas and oligodendroglial / oligoastrocytic gliomas **b.** Prognostic value of histological grade was independent of *IDH1/2* mutation status **c.** 1p/19q codeletion status **d.** and *TERT*p mutation status **e.** No prognostic association was found for histological grade among *EGFR* amplified gliomas **f.** OS, overall survival; mut, mutant; wt, wild-type.

Next, we investigated the influences of age, histological typing and molecular markers on the prognostic value of histological grade by co-evaluating histological grade and individual variables in survival analysis (Table 3, Fig. 3a to 3f). Histological grade could prognostically dichotomize patients of 50 years or younger (*p* < 0.0001) and the effect was marginal in patients above 50 years (*p* = 0.09) (Fig. 3a). Co-evaluating histological grade and type showed that the prognostic value of histological grade was independent of histological type (Fig. 3b). Grade III histology was associated with unfavorable prognosis in both astrocytic tumors (*p* < 0.0001) and oligodendroglial or oligoastrocytic tumors (*p* = 0.006). Prognostic stratification by histological grade in tumor groups defined by molecular markers was shown in Fig. 3c to 3f. Histological grade showed a good prognostic breakdown in *IDH1/2* mutated gliomas (*p* < 0.0001), *IDH1/2* wild-type gliomas (*p* < 0.0001), 1p/19q codeleted gliomas (*p* = 0.024), 1p/19q non-deleted gliomas (*p* < 0.0001), *TERT*p mutated gliomas (*p* < 0.0001) and *TERT*p wild-type gliomas (*p* < 0.0001), indicating that prognostic value of histological grade was independent of these three molecular markers. Co-evaluating histological grade and *EGFR* amplification status showed that grade could prognostically separate *EGFR* non-amplified lower-grade gliomas (*p* < 0.0001) but not *EGFR* amplified lower-grade gliomas (*p* < 0.811) (Fig. 3f). Though the number of *EGFR* amplified grade II gliomas was small (*n* = 6), it's worthwhile to note that this small subset of grade II tumors exhibited worse prognosis than *EGFR* non-amplified grade III gliomas (*p* = 0.006). This was consistent with the univariate analysis that the hazard ratio (HR) of amplified *EGFR* to non-amplified *EGFR* (HR = 9.082, 95% CI = 4.373–18.863) was greater than that of grade III to grade II histology (HR = 4.045, 95% CI = 2.629–6.223) (Table 2).

**Table 3:** Survival analysis of histological grade by clinical and molecular variables

	Median OS (years)		*p*
	Grade II	Grade III	
Age			
≤50 years	11.8	3.1	<0.0001
>50 years	4.4	2.1	0.09
Histological type			
Astrocytic	9.9	2.2	<0.0001
Oligodendroglial / Oligoastrocytic	11.8	3.1	0.006
*IDH1/2*			
Mutant	10.2	4.3	<0.0001
Wild-type	8.4	1.3	<0.0001
1p/19q			
Codeleted	NR	2	0.024
Non-codeleted	8.5	2.3	<0.0001
*TERT*p			
Mutant	11.8	2	<0.0001
Wild-type	9.9	2.3	<0.0001
*EGFR*			
Amplified	0.7	1.1	0.811
Non-amplified	11.8	2.5	<0.0001
Genetic signature			
IDHmut-OT	NR	10.2	0.004
IDHmut	7.8	4.3	0.0002
IDHwt	NR	1.5	0.0004
IDHwt-ET	1.3	0.4	0.04

OS, overall survival; NR, median survival not yet reached; IDHmut-OT, *IDH1/2* mutated and either 1p/19q codeleted or *TERT*p mutated; IDHmut, *IDH1/2* mutated only; IDHwt, *IDH1/2* wild-type, TERTp wild-type and *EGFR* non-amplified; IDHwt-ET, *IDH1/2* wild-type and either *TERT*p mutated or *EGFR* amplified.

### Survival analysis (Genetic signature)

**Figure 4:**
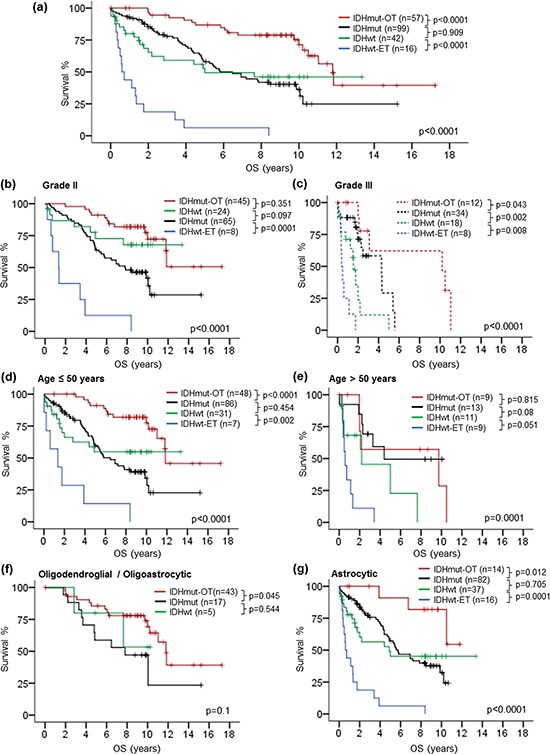
Kaplan-Meier survival analysis by genetic signature and co-evaluating genetic signature with clinical variables The genetic signature showed prognostic relevance across the whole cohort **a.** The genetic signature was associated with overall survival in grade II gliomas **b.** and grade III gliomas **c.** Prognostic association of the genetic signature was demonstrated in patients at or younger than 50 years **d.** as well as patients above 50 years **e.** The genetic signature exhibited prognostic relevance in astrocytic gliomas **g.** and trend in oligodendroglial / oligoastrocytic gliomas **f.** OS, overall survival; mut, mutant; wt, wild-type.

To further interrogate the survival data of our cohort and compare the prognostic value of molecular markers and histological grade, we assigned each tumor into genetic subgroups defined by combination of the molecular markers. We defined tumors with *IDH1/2* mutation and either 1p/19q codeletion or *TERT*p mutation as ‘IDHmut-OT’ gliomas, tumors with *IDH1/2* mutation only as ‘IDHmut’ gliomas, tumors with wild-type *IDH1/2* and either *TERT*p mutation or *EGFR* amplification as ‘IDHwt-ET’ gliomas, and tumors without any of the four molecular markers as ‘IDHwt’ gliomas. As shown in Fig. 4a, tumor subgroups defined by the genetic signature showed significant difference in overall survival across the whole cohort (*p* < 0.0001). Pair-wise comparison revealed that 57 IDHmut-OT gliomas showed longest overall survival compared to 99 IDHmut gliomas (*p* < 0.0001), 42 IDHwt gliomas (*p* = 0.002) and 16 IDHwt-ET gliomas (*p* < 0.0001). Comparing the survival between 99 IDHmut gliomas and 42 IDHwt gliomas, there was no statistical significance in overall survival between the two groups. Prognostic value of the genetic signature was further analyzed according to histological grade, patient age and tumor histology (Fig. 4b to 4g). The genetic signature showed statistical significance across both grade II (*p* < 0.0001) and grade III gliomas (*p* < 0.0001). In the 72 grade III gliomas, 12 IDHmut-OT tumors showed marginally favorable overall survival than 34 IDHmut tumors (*p* = 0.043), which in turn showed better survival than 18 IDHwt tumors (*p* = 0.002), followed by 8 IDHwt-ET tumors (*p* = 0.008) (Fig. 4c). In the 142 grade II gliomas, 45 IDHmut-OT tumors showed a trend of better prognosis than 24 IDHwt tumors in Kaplan-Meier's curve (*p* = 0.351), which in turn showed a trend of better prognosis than 65 IDHmut tumors (*p* = 0.097), followed by 8 IDHwt-ET tumors (*p* = 0.0001) (Fig. 4b). Analyzing according to patient age, the genetic signature showed a better stratification in patients of 50 years or younger (*p* < 0.0001) than in patients above 50 years (*p* = 0.0001) (Fig. 4d and 4e). The genetic signature was significant across 149 astrocytic gliomas (*p* < 0.0001) and marginally significant between 43 IDHmut-OT tumors and 17 IDHmut tumors with oligodendroglial or oligoastrocytic histology (*p* = 0.045) (Fig. 4f and 4g). In all subgroups of the cohort according to histological grade, age and astrocytic histology, tumors with IDHwt-ET signature showed poorest prognosis.

**Figure 5:**
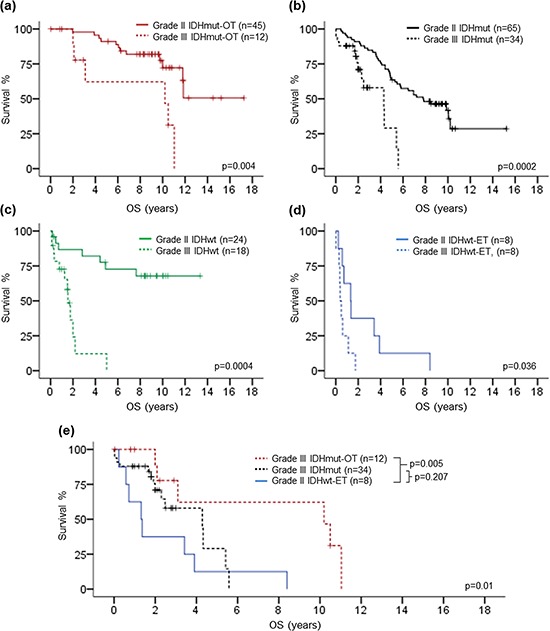
Kaplan-Meier survival analysis of histological grade in each of the genetic signature subgroup Histological grade showed prognostic association in IDHmut-OT gliomas **a.** IDHmut gliomas **b.** IDHwt gliomas **c.** as well as IDHwt-ET gliomas. Grade II IDHwt-ET gliomas demonstrated shorter overall survival than grade III IDHmut-OT gliomas and trend of shorter overall survival than grade III IDHmut gliomas **e.** OS, overall survival.

Prognostic value of histological grade in each of the genetic signature subgroups was also evaluated in order to investigate the influence of the genetic signature on prognostic value of histological grade (Fig. 5a to 5d). In all genetic signature subgroups, patients with grade II tumors had longer overall survival than those with grade III tumors, suggesting that histological grade was independent with the genetic signature in prognostication. Importantly, 8 grade II IDHwt-ET gliomas showed significantly shorter overall survival than 12 grade III IDHmut-OT gliomas (*p* = 0.005) and trend of shorter overall survival than 34 grade III IDHmut gliomas (*p* = 0.207) (Fig. 5e), suggesting that the genetic signature potentially had a more dominant prognostic value than histological grade in prognostication of lower-grade gliomas.

Multivariate analysis was performed by Cox proportional hazards model to evaluate the independent prognostic value of histological grade and genetic signature (Table 4). In the multivariate model adjusting for patient age, tumor histology and operation, both histological grade (*p* < 0.0001) and genetic signature (*p* < 0.0001) were independent prognostic factor for overall survival. The hazard ratio of grade III histology to grade II histology was 3.753 (95% CI = 2.214–6.363). For genetic signature, hazard ratio was greatest for IDHwt-ET tumors to IDHmut-OT tumors (HR = 19.491, 95% CI = 7.605–49.955, *p* < 0.0001), followed by IDHmut tumors to IDHmut-OT tumors (HR = 3.417, 95% CI = 1.704–6.852, *p* = 0.0005) and IDHwt tumors to IDHmut-OT tumors (HR = 2.958, 95% CI = 1.309–6.681, *p* = 0.009).

**Table 4:** Multivariate analysis of overall survival

	HR	[95% CI]	*p*
Age	1.027	[1.005–1.050]	0.015
Histological type			0.639
Astrocytic	1		
Oligodendroglial / Oligoastrocytic	0.869	[0.485–1.560]	
Operation			<0.0001
Total resection	0.382	[0.248–0.589]	
Non-total resection	1		
Histological grade			<0.0001
Grade II	1		
Grade III	3.753	[2.214–6.363]	
Genetic signature			<0.0001
IDHmut-OT	1		
IDHmut	3.417	[1.704–6.852]	0.0005
IDHwt	2.958	[1.309–6.681]	0.009
IDHwt-ET	19.491	[7.605–49.955]	<0.0001

HR, hazard ratio; 95% CI, 95% confidence interval; IDHmut-OT, *IDH1/2* mutated and either 1p/19q codeleted or *TERT*p mutated; IDHmut, *IDH1/2* mutated only; IDHwt, *IDH1/2* wild-type, *TERT*p wild-type and *EGFR* non-amplified; IDHwt-ET, *IDH1/2* wild-type and either *TERT*p mutated or *EGFR* amplified.

## DISCUSSION

Our study evaluated the influence of *IDH1/2* mutation, 1p/19q codeletion, *TERT*p mutation and *EGFR* amplification in the prognostic value of histological grade in a cohort of 214 cases of lower-grade gliomas. Co-evaluation of histological grade and molecular markers demonstrated the prognostic association of grading in lower-grade gliomas irrespective of the status of *IDH1/2*, 1p/19q and *TERT*p. Notably, no single molecular marker, including *IDH1/2* mutation, superseded histological grading in prognostication. *IDH1* mutation was found to have more dominant prognostic effect than histological grade in anaplastic astrocytomas and glioblastomas [27]. Our study provided complementary results as to the prognostic role of *IDH1* mutation relative to histological grade in lower-grade gliomas. A prognostic genetic signature was further constructed based on the biomarkers. Most favorable prognosis was observed in IDHmut-OT gliomas (median OS = 11.8 years), followed by IDHmut gliomas (median O*S* = 5.9 years) and IDHwt gliomas (median O*S* = 5 years), and IDHwt-ET gliomas showed the poorest prognosis (median OS = 0.6 year). Multivariate analysis further demonstrated the independent prognostic value of histological grading and genetic signature in lower-grade gliomas. Although histological grade exhibited prognostic association in each of the genetic subgroups, the overall survival of grade II IDHwt-ET gliomas was shorter than grade III gliomas harboring *IDH1/2* mutation, signifying combination genetic signature could potentially supersede histological grade in prognostication of lower-grade gliomas.

Prognostic stratification of diffuse gliomas by combinations of molecular markers was demonstrated by other research groups but the relative prognostic relevance between histological grading and marker combinations has not been intensively studied. Jiao and colleagues examined a cohort of grades II to IV diffuse gliomas for *IDH*, *ATRX*, *TP53*, *CIC* and *FUBP1* mutations as well as 1p/19q codeletion and classified the tumors into ‘I-CF’ (*IDH1/2* mutation with either 1p/19q loss, *CIC* mutation or *FUBP1* mutation), ‘I-A’ (*IDH1/2* mutation and *ATRX* mutation) and ‘I-X’ gliomas (neither I-CF nor I-A). I-CF group was associated with most favorable prognosis and I-X group was associated with poorest prognosis, with I-A group being in between. This I-CF/I-A/I-X genetic signature exhibited a better prognostic classification than histopathology in their cohort of grade III gliomas [34]. In another study, Wiestler and colleagues defined ‘molecular astrocytoma’, ‘molecular oligodendroglioma’ and ‘molecular glioblastoma’ by combinations of status of *IDH1/2*, 1p/19q and ATRX immunohistochemical expression and demonstrated that loss of ATRX expression refined classification of anaplastic gliomas by identifying a subgroup of *IDH* mutated anaplastic astrocytomas with better prognosis [35]. Mur and colleagues analyzed *IDH1/2* mutation, 1p/19q codeletion and ATRX expression and categorized the tumors into ‘I-CD’ (*IDH1/2* mutation, 1p/19q codeletion and ATRX positive expression), ‘I-A’ (*IDH1/2* mutation, absence of ATRX expression and intact 1p/19q), ‘I’ (*IDH1/2* mutation, intact 1p/19q and positive ATRX expression) and ‘NA’ (wild-type *IDH1/2*, intact 1p/19q, and positive ATRX expression) molecular groups. They showed that such molecular classification defined four prognostically distinct glioma groups irrespective of tumor diagnosis and grade [36]. On the other hand, combination of *TERT*p mutation with *IDH1/2* mutation was also shown to refine prognostic stratification of diffuse gliomas by several studies [19, 20, 23]. In these studies, four distinct prognostic groups were outlined by the two markers with *IDH1/2* mutated / *TERT*p mutated tumors having the most favorable survival, followed by *IDH1/2* mutated / *TERT*p wild-type tumors and *IDH1/2* wild-type / *TERT*p wild-type tumors, and *IDH1/2* wild-type / *TERT*p mutated tumors exhibiting the worst survival [19, 20, 23]. Our current study combined *IDH1/2* mutated tumors with either 1p/19q codeletion or *TERT*p mutation as IDHmut-OT gliomas since both of the markers were closely associated with oligodendroglial differentiation with the presence of *IDH1/2* mutation [19, 20, 23, 25, 26]. This combination allowed us to identify ‘molecular oligodendrogliomas’ with favorable prognosis, of which 9.4% of astrocytomas, 87.5% of oligodendrogliomas and 53.7% of oligoastrocytomas harbored the IDHmut-OT genetic signature. On the other end, IDH1/2 wild-type tumors with either *TERT*p mutation or *EGFR* amplification were defined as IDHwt-ET gliomas. Wild-type *IDH1/2* and mutated *TERT*p were identified in up to 80% of glioblastomas and was associated with poor prognosis in patients with the grade IV tumors [20, 22, 24]. A recent study by Labussiere and colleagues also demonstrated that combined analysis of *TERT*p, *EGFR* and *IDH1/2* defined distinct prognostic classes in glioblastomas, with the absence of both *EGFR* amplification and *TERT*p mutation associating with longer survival in patients with glioblastomas [22]. The prognostic value of IDHwt-ET genetic signature in lower-grade gliomas in our cohort was consistent with these studies. Patients with IDHwt-ET gliomas exhibited a similarly poor outcome (median OS = 0.6 year) compared with other lower-grade gliomas, suggesting that these patients may require more follow-up and treatment. Importantly, grade II IDHwt-ET gliomas had poorer prognosis than grade III IDHmut-OT gliomas and grade III IDHmut gliomas, indicating that IDHwt-ET genetic signature potentially supersedes histological grade in prognostication of lower-grade gliomas. Evaluation of *EGFR* amplification and *TERT*p mutation in a background of wild-type *IDH1/2* in lower-grade gliomas could help to identify aggressive tumors with seemingly low grade histology, facilitating a more precise prognostic estimation. Nevertheless, histological grade showed prognostic relevance in each genetic signature subgroup, suggesting its irreplaceable role in this biomarker-based stratification.

In our cohort, IDHmut gliomas and IDHwt gliomas demonstrated intermediate prognosis and there was no significant prognostic difference between these two groups. While *IDH1/2* mutation showed a wide prognostic split across the whole cohort (Fig. 1d), the lack of prognostic difference between IDHmut gliomas and IDHwt gliomas could be partially due to the elimination of the favorable prognostic effect of IDHmut-OT gliomas from the *IDH1/2* mutated tumors as well as the unfavorable prognostic effect of IDHwt-ET gliomas from the *IDH1/2* wild-type tumors, therefore bringing the two survival curves overlapping with each other (Fig. 4a). Alternatively, our data suggested that there existed an under-characterized subset of *IDH1/2* wild-type lower-grade gliomas with comparable survival to *IDH1/2* mutated gliomas lacking 1p/19q codeletion and *TERT*p mutation. The data further suggested that not all *IDH1/2* wild-type lower grade gliomas are uniformly aggressive and additional markers including *TERT*p mutation and *EGFR* amplification should be examined in the clinical management of lower-grade gliomas with wild-type *IDH1/2*. This was corroborated by two recent studies by Weller *et al.* and Olar *et al*. [37, 38]. Weller and colleagues conducted comprehensive genomic and transcriptomic profiling in grades II and III gliomas to determine the prognostic utility of molecular profiling in the tumor entity. The authors demonstrated the existence of a genomic group of *IDH1/2* wild-type lower-grade gliomas (Group IV) lacking glioblastoma-like genomic aberrations such as 7q gains and 10q losses. This group of tumors had intermediate prognosis as *IDH1/2* mutated tumors lacking 1p/19q codeletion (Group II and III), indicating that merely wild-type *IDH1/2* status may not be invariably associated with poor clinical outcome and additional glioblastoma-like molecular alterations are required to prognosticate a particularly unfavorable survival [38]. Olar and colleagues investigated a large cohort of lower-grade gliomas to determine the prognostic role of histological grade and mitotic index following stratification by *IDH1/2* mutation. The authors showed that combining *IDH1/2* mutation, 1p/19q codeletion and mitotic index (4/1000 tumor cells as cut-off) could stratify lower-grade gliomas into four prognostic groups, with 1p/19q codeletion identified tumors with better prognosis in *IDH1/2* mutated group and mitotic index >4 identified tumors with poorer prognosis in *IDH1/2* wild-type group [37]. Notably, there was a portion of *IDH1/2* wild-type / mitotic inde*x* = 0–4 tumors showing comparable survival with *IDH1/2* wild type / 1p/19q non-codeleted tumors. The results provided further evidence that additional investigations should be carried out in lower-grade gliomas with wild-type *IDH1/2* for better prognostication.

The prognosis of IDHwt-ET lower-grade gliomas was poor and comparable to glioblastomas. Among the 16 IDHwt-ET gliomas, nine cases harbored *EGFR* amplification as demonstrated by FISH (Supplementary Fig. S1 & Table S1). The definitions of *EGFR* amplification varied in different cancer types and even within diffuse gliomas, the cut-off criteria varied in different studies [31, 39–47]. This was partially because the pattern of *EGFR* gain or amplification could not be seen normally in non-neoplastic brain tissues and therefore the cut-off could not be determined by statistical methods using mean plus three standard deviations as in deletion detection [31, 44, 48, 49]. In this study, all *EGFR* amplified cases demonstrated a pattern of clusters of tumor cells with high level amplification (Supplementary Fig. S1). Presence of *EGFR* amplification and involvement of deep midline structures (including corpus callosum, thalamus, basal ganglia, ventricles and midbrain) in four cases suggested the possibility that these tumors are under-sampled high-grade gliomas [43, 50]. Nevertheless, examination of the biomarkers represented a satisfactory way of removing sampling issues in these aggressive tumors. We also reviewed the pre-operative radiological reports still available in our hospital system in four available cases and contrast enhancement was demonstrated in one anaplastic astrocytoma while others presented as hypodense lesions. Although we could not identify any concrete evidence within the available retrospective clinical data that the *EGFR* amplified tumors involved more than two cerebral lobes with extraordinary diffuse infiltration, i.e. gliomatosis cerebri [51, 52], the possibility that gliomatosis cerebri presented with low grade histology but harboring high grade molecular aberrations could not be excluded. Notably, *EGFR* amplification was not detected in two large gliomatosis cerebri series [53, 54], though 7q gain as detected by comparative genomic hybridization was found to be a poor prognostic factor in gliomatosis cerebri [55].

In summary, our study demonstrates the independent prognostic values of histological grading and genetic signature based on *IDH1/2* mutation, 1p/19q codeletion, *TERT*p mutation and *EGFR* amplification. The biomarker-based stratification provides complementary prognostic information potentially superseding histology and refines the diagnostic and prognostic classification of lower-grade gliomas.

## MATERIALS AND METHODS

### Patients, tissue samples and clinico-pathological data

A total of 214 cases of lower-grade gliomas diagnosed between 1990 and 2012 with formalin-fixed paraffin embedded tissue available were retrieved from the tissue archive of Department of Anatomical and Cellular Pathology, Prince of Wales Hospital (Hong Kong) and Department of Neurosurgery, Huashan Hospital (Shanghai). All tumor sections were stained with haematoxylin and eosin (H&E) and reviewed carefully by two senior neuropathologists (H.K. Ng and Y. Wang). Histologic classification and grading are according to the World Health Organization (WHO) 2007 classification [28]. For diagnosis of Grade III astrocytoma, the criteria were cellularity, atypia and mitosis. For diagnosis of Grade III oligodendroglioma, the criteria are also cellularity, atypia and mitosis, and/or with microvascular proliferation or necrosis. Necrosis and endothelial proliferation are carefully looked for and those cases are excluded. Patient demographics and clinical follow-up data were retrieved from the respective institutional medical record systems. The cohort was overlapped with previous studies [19, 29]. The study was approved by the New Territories East Cluster-Chinese University of Hong Kong Ethics Committee and Shanghai Huashan Hospital Ethics Committee.

### Fluorescence *in situ* hybridization for chromosome 1p/19q codeletion and *EGFR* amplification

Chromosome 1p and 19q codeletion and *EGFR* amplification were evaluated by fluorescence *in situ* hybridization [19, 29, 30]. The loci examined for 1p and 19q were 1p36.3 (RP11–62M23 labeled red) / 1q25.3-q31.1 (RP11–162L13 labeled green) and 19q13.3 (CTD-2571L23 labeled red)/19p12 (RP11–420K14 labeled green), respectively. Locus-specific probes for chromosome 1 and 19 were generated from bacterial artificial chromosome clones using nick translation with the presence of Spectrum Orange deoxyuridine triphosphate (dUTP) or Spectrum Green deoxyuridine triphosphate (dUTP). The labelled probes were then mixed with cot-1 DNA (Life Technologies) in Hybrisol VI solution (Appligene Oncor, Graffenstaden, France). Vysis EGFR/CEP7 FISH Probe Kit (Abbott Laboratories, Illinois, USA) was used to examine *EGFR* and the loci interrogated were 7p12 (*EGFR*) / 7p11.1-q11.1 (CEP7). Four-μm thick formalin-fixed, paraffin embedded tissue sections were deparaffinized by xylene and treated with 1 M sodium thiocyanate at 80°C for 10 minutes, followed with tissue digestion by pepsin solution at 37°C for 20 to 30 minutes. Sections were then rinsed in milli-Q water and dehydrated. The FISH probes were applied to the digested tissue sections and denature. The sections were incubated at 37°C overnight for 16 hours. After overnight hybridization, sections were washed in 1.5 M urea in 0.1X saline sodium citrate at 48°C for 30 minutes, followed by 2X saline sodium citrate at 48°C for 5 minutes. Sections were then counterstained with Vectashield mounting medium containing 4′,6-diamidino-2-phenylindole (DAPI; Vector Laboratories) and evaluated under a Zeiss Axioplan fluorescence microscope (Carl Zeiss Microscopy LLC, NY, USA). Fluorescent signals in at least 100 non-overlapping nuclei were evaluated. 1p loss or 19q loss were defined as more than 50% of counted nuclei showing target (red) to reference (green) signal ratio of 1:2. *EGFR* amplification was defined as more than 5% tumor cells showing target (red) to reference (green) signal >2 [31].

### Mutational analysis for *IDH1*, *IDH2* and *TERT* promoter

Mutations of *IDH1*, *IDH2* and *TERT*p were detected by direct sequencing as described previously [19, 29, 32, 33]. In brief, tissues from representative area with tumor content of at least 70% were scrapped off from deparaffinized sections and treated in 10 mM Tris-HCl buffer (pH8.5) with proteinase K at a final concentration of 2 μg/μl at 55°C for 2 to 18 hours and then 98°C for 10 minutes. The cell lysate was centrifuged and supernatant was collected and used for subsequent PCR amplification. Primer pairs spanning the mutation hotspots of *IDH1* (codon 132), *IDH2* (codon 172) and *TERT*p [chr5, 1,295,228 (C228T) and 1,295,250 (C250T)] were used for PCR amplification and primer sequences were shown in Supplementary Table S2. For PCR of *IDH1* and *IDH2*, DNA amplification was conducted in a 10-μl reaction volume containing 1μl of cell lysate, 10 mM Tris-HCl (pH 8.3), 50 mM KCl, 2.5 mM MgCl2, 0.2 mM of each deoxyribonucleoside triphosphate, 0.4 μM of each primer and 0.2U of AmpliTaq Gold DNA polymerase (Life Technologies Corporation, Hong Kong), and was incubated at 95°C for 10 minutes, followed by 40–45 cycles of 95°C for 20 seconds, 60°C for 20 seconds and 72°C for 30 seconds, and a final extension of 72°C for 1 minute. For amplification of *TERT*p, PCR was performed in 10μl reaction mixture containing 1μl of cell lysate, 0.3 mM of each dNTP, 2.5 mM MgCl2, 0.3 μM of each primer and 0.2U of KAPA HiFi HotStart DNA Polymerase (Kapa Biosystem Inc., Wilmington, USA). The reaction mixture was incubated at 95°C for 5 minutes, followed by 40–45 cycles of 98°C for 20 seconds, 68°C for 15 seconds and 72°C for 30 seconds, and a final extension of 72°C for 1 minute. PCR products were then treated with exonuclease I and alkaline phosphatase (Takara, Japan) at 37°C for 15 minutes and then at 80°C for 15 minutes. Sequencing was performed using BigDye Terminator Cycle Sequencing kit v1.1 (Life Technologies). The products were resolved in Genetic Analyzer 3130xl and analyzed by Sequencing Analysis software.

### Statistical analysis

Statistical analysis was conducted using IBM SPSS Statistics 20 (IBM Corporation, NY, USA). Association between molecular markers and clinical parameters were examined by Chi square test or Fisher's exact test, whichever was appropriate. Overall survival (OS) was defined as the time between diagnosis and death or last follow-up. Survival curves were constructed by Kaplan-Meier method and survival distribution between groups was compared by Log-rank test. Multivariate analysis was performed by Cox proportional hazards model. *P*-value of <0.05 (two sided) was considered statistically significant.

## SUPPLEMENTARY FIGURE AND TABLES


