# Supplementary material for: Combination genetic signature stratifies lower-grade gliomas better than histological grade
Source: Oncotarget. 2015 Aug 11;6(25):20885–901. doi: 10.18632/oncotarget.4928 (PMC4673237; doi:10.18632/oncotarget.4928)
Supplement: Supplementary file 1 [file oncotarget-06-20885-s001.pdf]

SUPPLEMENTARY FIGURE AND TABLES

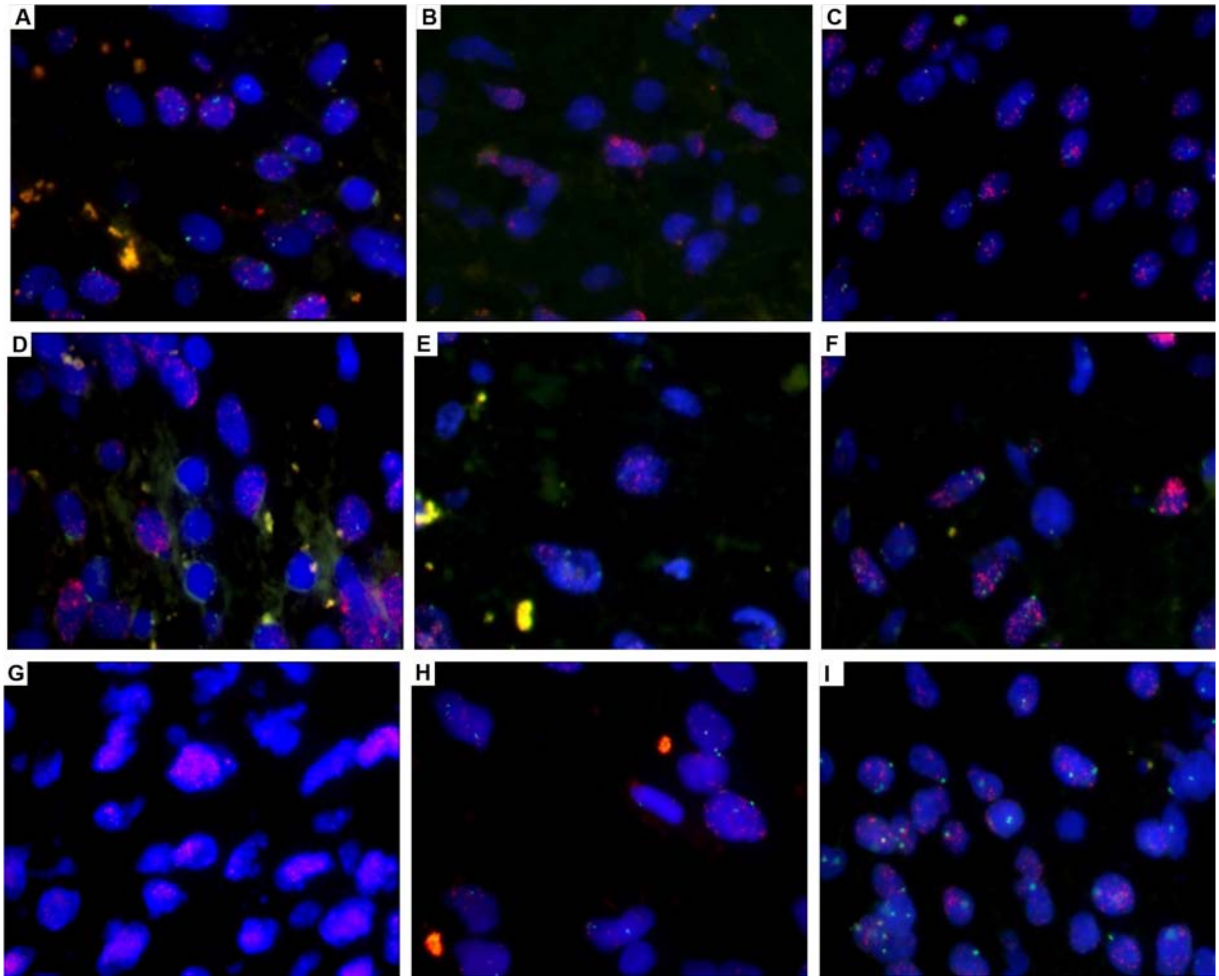

Supplementary Figure S1: Representative fluorescence *in-situ* hybridization micrograph showing clusters of tumor cells with *EGFR* gene amplification (red) in diffuse astrocytomas A to F. and anaplastic astrocytomas G to I.

Supplementary Table S1: Clinical data of nine patients with *EGFR* amplified lower-grade gliomas

Age/sex	Diagnosis	Tumor location	Pre-operative radiological finding	Operation	Radiotherapy	Chemotherapy	Status	OS (years)
46 / F	AII	left frontal	Contrast CT - Hypodense non-enhancing lesion in left frontal parasagital region	Bx	Yes	Yes	Dead	1.4
57 / F	AII	right frontal	Plain CT - Subtle hypodense lesion over right frontal parafalcine region with diminished grey-white matter differentiation	Bx	Yes	No	Dead	1.3
51 / F	AII	corpus callosum	N/A	N/A	N/A	N/A	Dead	0.7
64 / M	AII	right parietal, corpus callosum, periventricular	N/A	PR	Yes	No	Dead	0.6
48 / M	AII	left thalamus, basal ganglion, midbrain	N/A	Bx	No	No	Dead	0.2
46 / F	AII	left parietal	N/A	Bx	Yes	No	Dead	3.9
48 / F	AAIII	intraventricular	Contrast CT - intraventricular heterogeneous tumor	Bx	No	No	Dead	0.01
54 / F	AAIII	right frontal	MRI - T2 diffuse signal in right frontal region with gyral increase hyperintensity, no contrast enhancement	Bx	Yes	No	Dead	1.1
47 / F	AAIII	left temporal	N/A	GTR	Yes	Yes	Dead	1.8

GTR, gross total resection; PR, partial resection; Bx, biopsy; OS, overall survival; N/A, data not available

Supplementary Table S2: Primers used in PCR of *IDH1*, *IDH2* and *TERT*p

	Sense	Antisense	Product size
IDH1	CGGTCTTCAGAGAAGCCATT	CACATTATTGCCAACATGAC	122 bp
IDH2	AGCCCATCATCTGCAAAAAC	CTAGGCGAGGAGCTCCAGT	150 bp
***TERT*p**	GTCCTGCCCCTTCACCTT	CAGCGCTGCCTGAAACTC	163 bp
